# Treatment and Rehabilitation of a Patient with Neuromyelitis Optica Spectrum Disorder-Induced Complete Spinal Cord Injury Following COVID-19 Vaccination: A Case Report

**DOI:** 10.3390/jcm13041175

**Published:** 2024-02-19

**Authors:** Jun-Sang Han, Seong-Mun Ryu, Young-Hwan Lim, Ae-Ryoung Kim, Tae-Du Jung

**Affiliations:** 1 Department of Rehabilitation Medicine, Kyungpook National University Hospital, Daegu 41944, Republic of Korea; goodream123@gmail.com (J.-S.H.); po01086@naver.com (S.-M.R.); limyoungh@naver.com (Y.-H.L.); ryoung20@hanmail.net (A.-R.K.); 2 Department of Rehabilitation Medicine, School of Medicine, Kyungpook National University, Daegu 41944, Republic of Korea; 3 Department of Rehabilitation Medicine, Kyungpook National University Chilgok Hospital, Daegu 41404, Republic of Korea

**Keywords:** autoimmune disorders, neuromyelitis optica spectrum disorder, COVID-19 vaccination, spinal cord injury, rehabilitation

## Abstract

Neuromyelitis optica spectrum disease (NMOSD) is a rare autoimmune disorder of the central nervous system characterized by optic neuritis, myelitis, or brain lesions. Its symptoms overlap with those of multiple sclerosis (MS), making a diagnosis of NMOSD challenging. Here, we report a rare case of NMOSD-induced complete spinal cord injury following COVID-19 vaccination. A 52-year-old female patient developed NMOSD-induced complete spinal cord injury after receiving their third dose of the Pfizer–BioNTech COVID-19 vaccine (BNT162b2). Despite the initial diagnosis of complete spinal cord injury, the patient underwent intensive treatment, including rituximab therapy and rehabilitation. As a result, she made a full recovery and transitioned from the ASIA Impairment Scale(AIS)-A to AIS-E. The remarkable neurological recovery from complete spinal cord injury to functional independence highlights the efficacy of a comprehensive treatment approach. In addition, this case emphasizes the need to recognize NMOSD as a potential adverse outcome of COVID-19 vaccination and emphasizes the importance of early diagnosis, timely intervention, and thorough rehabilitation for optimizing patient results. Further case reports and studies are needed to investigate the association between COVID-19 vaccination and the occurrence of NMOSD.

## 1. Introduction

Neuromyelitis optica spectrum disorder (NMOSD) is a neuroinflammatory disorder of the central nervous system characterized by recurrent optic neuritis, myelitis, or brain lesions. Initially, NMOSD was classified as a subtype of multiple sclerosis (MS) owing to its clinical similarities. NMOSD and MS present with similar clinical manifestations and habitual relapses, complicating the diagnosis of NMOSD; however, some MS treatments can exacerbate NMOSD [1,2]. Therefore, differential and accurate NMOSD diagnosis is crucial. The identification of aquaporin-4 IgG (AQP4-IgG) in patients with NMOSD demonstrates its distinction from MS. In 2015, the diagnostic criteria for NMOSD were revised to include the presence or absence of AQP4-IgG [3]. The discovery of AQP4-IgG has advanced our understanding of the pathogenesis of NMOSD and has facilitated the use of B-cell-directed therapies [3,4,5,6]. However, several cohort studies have revealed that a subset of patients with NMOSD tests were negative for AQP4-IgG [7,8,9]. Recent evidence suggests that some patients with NMOSD are associated with myelin oligodendrocyte glycoprotein IgG (MOG-IgG) [10,11,12]. With the appropriate management of acute attacks using various immunosuppressive therapies, patients with NMOSD generally have a favorable prognosis [13,14,15].

The exact cause of NMOSD is not yet known, but new evidence suggests that viral infections may play a potential role in triggering the development of NMOSD [16,17]. The ongoing global pandemic is caused by severe acute respiratory syndrome coronavirus 2 (SARS-CoV-2), resulting in the novel coronavirus disease known as COVID-19 [18]. Following the development of several COVID-19 vaccines, various adverse effects have been reported post-vaccination [19].

NMOSD is a recognized cause of non-traumatic spinal cord injury (NTSCI) [20]. The International Standards for Neurological Classification of Spinal Cord Injury (ISNCSCI) is widely utilized for the assessment of the severity and level of spinal cord injury (SCI). The prognosis of individuals diagnosed using the ASIA Impairment Scale (AIS) is generally poor [21]. During the initial neurological evaluation, patients with NTSCI often present with higher AIS grades and less neurological damage than those with traumatic SCI [22].

Here, we present a case study involving a patient with NTSCI who was initially diagnosed with complete AIS-A SCI. The patient was subsequently diagnosed with NMOSD following the COVID-19 vaccination. Through appropriate treatment and a rigorous 7-month rehabilitation program, the patient recovered significantly and transitioned from AIS-A to AIS-E.

## 2. Case Presentation

**Figure 1:**
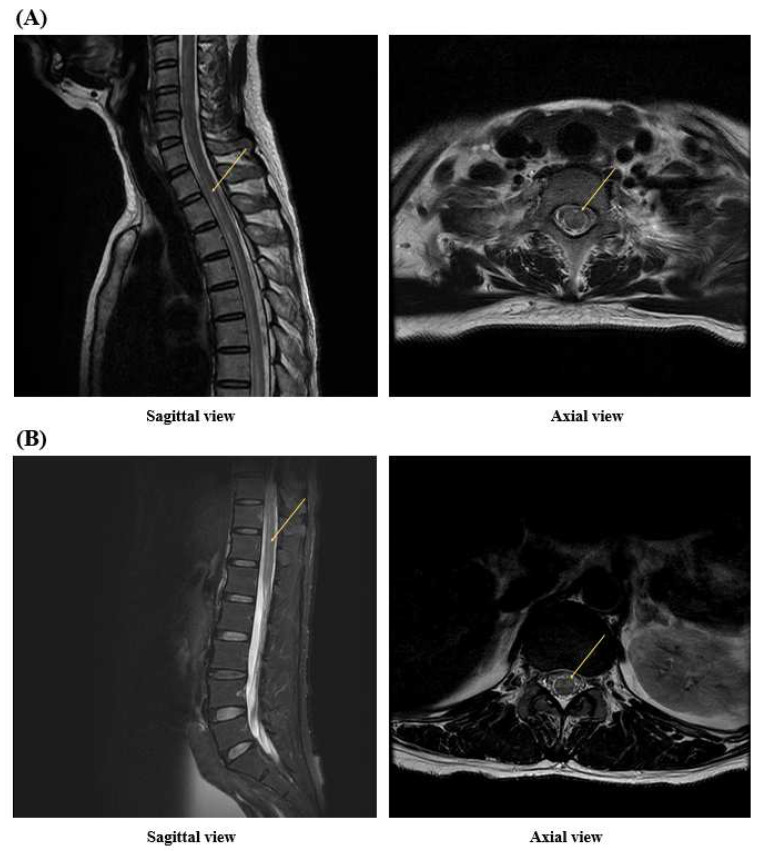
In (**A**), arrows indicates high signal intensity starting from the T2 vertebral body segment in the T2-weighted MRI image. In (**B**), arrows indicates high signal intensity in the T12 vertebral body segment on the T2-weighted MRI image. Longitudinally extensive transverse myelitis (LETM) involves three or more vertebral body segments. In the patient above, the lesion started from the T2 vertebral body segment and spanned 12 segments up to the T12 vertebral body segment. This is an important imaging finding in diagnosing NMOSD.

**Figure 2:**
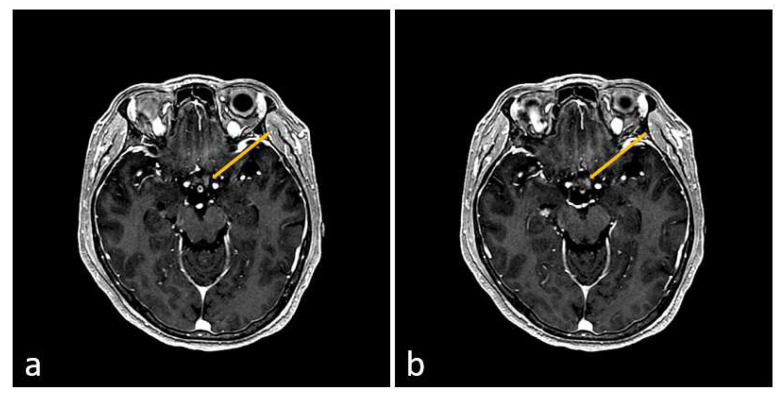
In (**a**), the arrow indicates high signal intensity of the left optic nerve on the T1 contrast-enhanced image of the brain MRI, and in (**b**), the arrow indicates high signal intensity of the optic chiasm on the T1 contrast-enhanced image of the brain MRI. These are important imaging findings in the diagnosis of NMOSD.

A 52-year-old female patient presented to the emergency department 10 days after receiving their third dose of the Pfizer–BioNTech COVID-19 vaccine (BNT162b2). She complained of pain in both eyes, numbness in both legs, difficulty walking, and dysuria. The patient had no history of similar episodes. Cognitive impairment or loss of consciousness was not observed. Polymerase chain reaction (PCR) tests performed upon admission to the neurology department were negative for COVID-19, adenovirus, the Epstein–Barr virus, cytomegalovirus, and human immunodeficiency virus. Cerebrospinal fluid analysis (CSF) was unremarkable, except for an increase in the total protein to 101.3 mg/dL. In the T2-weighted image of spinal MRI, an intramedullary high signal intensity lesion extending from the T2 vertebral body segment level to the T12 vertebral body segment was confirmed (Figure 1). On the second day of hospitalization, the muscle weakness in both lower extremities worsened, and no voluntary movements were observed. Brain MRI showed high signal intensity lesions in the optic chiasm and optic nerve (Figure 2), and VEP testing confirmed central conduction delay; therefore, the disease was diagnosed as NMOSD according to the 2015 diagnostic criteria for NMOSD [3].

Antibody tests performed at the time of admission showed that AQP4-IgG was negative and MOG-IgG was positive. Intravenous methylprednisolone (IVMP) pulse therapy was initiated on the day of admission and continued for 5 days, followed by oral steroid therapy. On day 4 of hospitalization, plasmapheresis was performed for 5 days. On day 10, rituximab therapy was initiated, and a total of six doses were administered monthly. On day 20, the patient was transferred to the Department of Rehabilitation Medicine. Rehabilitation treatment was conducted 5 days a week. Two sessions of physical therapy and one session of daily life training were conducted per day; the duration of each session was 30 min. In the physical examination performed after the transfer to the Department of Rehabilitation Medicine, the strength of both lower extremities was assessed as zero according to the manual muscle test (MMT), and there was no voluntary anal contraction. Deep tendon reflexes (DTRs) of both lower extremities were enhanced, and Babinski reflexes were positive. The patient was unable to feel a perianal sense or deep anal pressure. Consequently, the AIS of the ISNCSCI was evaluated as AIS-A at the time of transfer to the Department of Rehabilitation Medicine. On day 25, compound muscle action potential (CMAP) and sensory nerve action potential (SNAP) were normal in the nerve conduction study (NCS), but motor unit action potential (MUAP) and muscle recruitment in both lower extremities were not observed during the electromyography (EMG) examination. Through this, it was confirmed that there was no voluntary muscle contraction in the lower extremity.

Trunk stability was reduced, and ambulation was not possible. Core muscle strength training was performed to improve the trunk balance. Functional electrical stimulation was applied to induce muscle contractions in both lower extremities. On day 70, spontaneous muscle contractions of the lower extremities were observed. Follow-up EMG showed MUAP and recruitment in both lower extremities. Thereafter, standing exercises using a standing incline and table were performed. As rituximab treatment progressed, muscle strength gradually recovered, and standing training with Knee–Ankle–Foot Orthosis (KAFO) was initially performed to promote muscle activation during standing. Four months after hospitalization, she was able to walk with some support, at which point she proceeded with gait continuation training on a treadmill and stair climbing. The patient was discharged 90 days after inpatient rehabilitation treatment, and after discharge, outpatient treatment was performed 3 days a week. One session of physical therapy and one session of daily life movement training were conducted daily.

**Figure 3:**
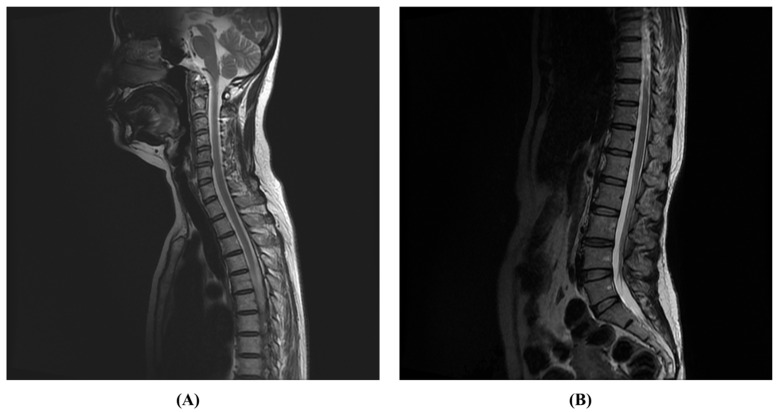
These figures are T2-weighted images of a spine MRI performed 7 months after onset. Compared to the previous MRI, it can be seen that longitudinally extensive transverse myelitis (LETM) findings from the T2 vertebral body segment to the T12 vertebral body segment disappeared. (**A**): Sagittal view on T2-weighted MRI imaging at the cervical and thoracic spine level, (**B**): Sagittal view on T2-weighted MRI imaging at the thoracic and lumbar spine level.

After 6 months of rehabilitation, the patient’s Berg balance scale (BBS) score improved from 3 to 56, and her modified Barthel index (MBI) score improved from 23 to 98. The expanded disability status scale (EDSS) score, initially measured by the neurology department, improved from 8.5 to 1.0. She was able to walk independently and experienced no significant discomfort in her daily activities other than numbness in both legs (Table 1). Seven months after onset, she was able to resume her previous life, including returning to work, and a follow-up spine MRI showed that the LETM seen on the initial spine MRI had disappeared (Figure 3).

## 3. Discussion

NMOSD is a rare autoimmune disease that primarily affects the optic nerve and the spinal cord [23]. NMOSD can occur at various ages; however, the average age of onset is 39 years, which is typically approximately 10 years older than that of MS [24]. It is crucial for NMOSD to be differentiated from acute disseminated encephalomyelitis, MS, the Guillian–Barre syndrome, and neuro-Behçet’s disease. The diagnosis of NMOSD can be challenging because the condition can mimic other neurological disorders, and there is no definitive diagnostic test [25]. However, key clinical features and diagnostic tests can help identify NMOSD [3]. The diagnostic criteria for NMOSD have been continuously revised based on new research findings. The most recent international consensus diagnostic criteria, published in 2015, include clinical and imaging features, serological testing for AQP4-IgG, and exclusion criteria for alternative diagnoses [3]. In addition to traditional optic neuritis and acute myelitis, the new diagnostic criteria established area postrema syndrome, acute brainstem syndrome, narcolepsy or acute diencephalon lesions, and symptomatic cerebral lesions as “core clinical features”; this allowed for the diagnosis of antibody-positive optic neuromyelitis if they had one of these six features and were antibody-positive with other diseases excluded [3]. In the present case, AQP4-IgG was not detected; however, MOG-IgG was detected, and the patient exhibited longitudinally extensive transverse myelitis (LETM) symptoms. In addition, optic neuritis was identified on a brain MRI during the clinical core symptoms; therefore, MOG-IgG-positive NMOSD without AQP4-IgG was diagnosed.

There is no consensus regarding the optimal treatment for NMOSD; however, various immunosuppressive agents have been used with varying degrees of success [26]. Corticosteroids are commonly used as the first-line treatment for acute exacerbations, but their side effects limit their long-term use [27]. Plasmapheresis or intravenous immunoglobulin (IVIG) may be considered for refractory cases [27]. Recent studies have shown that B cell-targeting agents—such as rituximab, ocrelizumab, and ofatumumab—are effective for treating MOG-IgG-positive NMOSD. These agents selectively target B cells, which are believed to play a role in disease [28]. There is also emerging evidence that complement inhibitors such as eculizumab and labulizumab may be effective in treating MOG-IgG-positive NMOSD. These agents target the complement system, which is involved in disease pathophysiology [28,29]. Our patient was treated with IVMP pulse therapy followed by 5 days of plasmapheresis and six cycles of rituximab therapy at 1-month intervals; the patient had a favorable prognosis.

The AIS provides a standardized framework for describing and communicating the extent of nerve damage caused. It helps clinicians, researchers, and medical professionals understand the severity of injuries, predict potential recovery, and guide treatment decisions. The AIS comprises five grades (A–E) that describe the level of sensory and motor impairment in individuals with SCI [30]. Individuals with complete spinal cord injury (AIS-A) generally have a poor prognosis. This usually indicates that there is little chance of significant neurological recovery. Complete damage represents the complete loss of sensory and motor functions, and prospects for the recovery of neurological function are usually limited. The probability of recovery for patients with AIS-A to AIS-D ranges from 2 to 5%, and it is even rarer for an AIS-A patient to recover to AIS-E [22,31,32]. However, rehabilitation can still play an important role in optimizing patients’ quality of life and functional independence.

The patient, in this case, was evaluated as AIS-A at the time of transfer to the Department of Rehabilitation Medicine. However, after appropriate intervention and 10 months of rehabilitation treatment, she recovered to AIS-E and returned to normal daily life. Several studies have reported that AQP4-IgG seropositivity in late-onset NMOSD (age ≥ 50 years) is associated with severe disability and high final EDSS scores [33,34]. Additionally, a worse prognosis is observed in patients with double-seronegative (MOG-IgG and AQP4-IgG) late-onset NMOSD than with AQP4-IgG-positive NMOSD, emphasizing the importance of serum status [33]. Since the patient was only MOG-IgG-positive, it was thought that the prognosis would be better than that of a patient with APQ4-IgG-positive or both MOG-IgG and AQP4-IgG-positive NMOSD. Furthermore, early NMOSD diagnosis and the administration of IVMP pulse therapy, plasmapheresis, and rituximab treatment at the appropriate time without delay in treatment might have played a major role in the patient’s recovery. Moreover, as the patient’s motor function recovered, appropriate rehabilitation treatment was concurrently performed; thus, independent walking was possible 4 months after symptom onset, and the patient was able to return to daily life and work after 7 months.

With the recent development of various COVID-19 vaccines, there is growing concern regarding the impact of vaccines on organs. The most common adverse events were localized pain at the injection site, low-grade fever, fatigue, myalgia, chills, and arthralgia, with rare cases of anaphylactic shock [19]. Recently, neuro-inflammatory disorders, such as NMOSD and MS, have been reported [35,36,37]. Although its pathogenesis is not clearly understood, human antigens and SARS-CoV-2 molecules can exhibit similar molecular properties, potentially causing autoimmune diseases in vaccine recipients [38]. Twenty-one human tissue antigens were cross-reactive with SARS-CoV-2 antibodies, offering a potential explanation for the observed autoimmunity resulting from SARS-CoV-2 mRNA vaccines that affect various systems, including the gastrointestinal tract, cardiovascular system, and nervous system [38].

In a recently published systematic review, eight patients with NMOSD, which developed after COVID-19 vaccination, were included. In all eight cases, five (62.5%) patients were female, and the mean (SD) age was 41.7 (12.4) years. Additionally, the mean (SD) interval between the injection of the COVID-19 vaccination and the first symptoms of NMOSD after the 1st and 2nd doses was 10.2 (5.3) and 7.6 (11.5) days, respectively. In total, 87.5% of patients received IVMP pulse therapy, 37.5% underwent plasmapheresis, and 37.5% received rituximab. Six out of eight NMOSD patients tested positive for AQP4-IgG [37]. When comparing our case with those in systematic reviews, the patient was female, and the age was 52 years, which is older than the average of 41.7 years. IVMP pulse therapy and plasmapheresis were used, and rituximab was additionally administered. In the systematic review, there were no reports of NMOSD occurring after the 3rd dose of vaccination, but in our case, NMOSD occurred 10 days after receiving the 3rd dose of the Pfizer–BioNTech COVID-19 vaccine (BNT162b2). This did not show much difference from the time interval until the first symptoms of NMOSD occurred after the first and second vaccinations. Additionally, unlike the eight cases, our case is the only one in which AQP4-IgG was negative and MOG-IgG was positive.

In the previous systematic review, some limitations were identified, including the small number of cases that might not be representative of the population and the lack of available data for all COVID-19 vaccines [37]. Therefore, additional case reports and further research are required. We believe that our case report provides important data for studying the relationship between COVID-19 vaccines and NMOSD.

## 4. Conclusions

In conclusion, clinicians should not overlook the possibility of NMOSD after COVID-19 vaccination and should promptly diagnose it through imaging, physical, and serological examinations. A good prognosis can be expected if appropriate intervention and rehabilitation treatments are combined with early diagnosis. Further research is required to understand the relationship between the COVID-19 vaccination and NMOSD.

## Figures and Tables

**Table 1:** Outcome measurements during various time points in patient’s hospital course.

		Admission	HD at 20 Days (Transfer to RM)	HD at 2 Months	HD at 4 Months (Discharge)	3 Months after Discharge
BBS		NC	3	4	54	56
K-MBI		NC	23	30	49	98
AIS		NC	A	B	D	E
MRS		5	5	5	2	0
EDSS		8.5	NC	8	NC	1
Dynamic test	TUG	NC	NT	NT	28.17 s	8.30 s
	10 m walk test	NC	NT	NT	28.95 s	7.99 s
	6 min walk test	NC	NT	NT	90 m	415 m

RM: Rehabilitation Medicine; BBS: Berg balance scale; K-MBI: Korean version of the modified Barthel index; AIS: ASIA Impairment Scale; MRS: modified Rankin scale; EDSS: expanded disability status scale; TUG: timed up and go; HD: hospital day; NT, not tested, NC, not checked.

## Data Availability

The datasets used during the current study are available from the corresponding author on reasonable request.
